# The response to thermospermine is fine-tuned by the balance between SAC51 and LHW family proteins in *Arabidopsis thaliana*

**DOI:** 10.3389/fpls.2025.1654744

**Published:** 2025-09-02

**Authors:** Yao Xu, Mitsuru Saraumi, Tomohiko Toyoshima, Hiroyasu Motose, Taku Takahashi

**Affiliations:** Graduate School of Environmental, Life, Natural Science and Technology, Okayama University, Okayama, Japan

**Keywords:** *Arabidopsis thaliana*, LHW family, SAC51 family, thermospermine, xylem

## Abstract

Thermospermine negatively regulates xylem formation. In *Arabidopsis*, *SAC51* and *SACL3*, members of the *SAC51* gene family encoding basic loop-helix-loop (bHLH) proteins play a key role in this regulation. These mRNAs contain an upstream open-reading-frame (uORF) that is highly conserved across species, and its inhibitory effect on the main ORF translation is alleviated by thermospermine. A double knockout of *SAC51* and *SACL3* results in thermospermine insensitivity at high concentrations that normally inhibit xylem formation and shoot growth in the wild type. Conversely, uORF mutants of *SAC51*, *SACL3*, and *SACL1* suppress the excessive xylem formation and dwarf phenotype of *acl5*, a mutant defective in thermospermine biosynthesis. In this study, we generated genome-edited uORF mutants of *SACL2* and confirmed that they partially recover the *acl5* phenotype. All uORF mutants exhibited increased sensitivity to thermospermine. SACL3 represses the function of LHW, a key bHLH transcription factor required for xylem proliferation, through direct interaction. We found that the *lhw* mutant is also hypersensitive to thermospermine, while this sensitivity was suppressed by the *sac51 sacl3* double knockout. Yeast two-hybrid assays demonstrated that all four SAC51 family members interact with LHW and its family members. These findings suggest that overaccumulation of SAC51 family proteins leads to thermospermine hypersensitivity by repressing the function of LHW family proteins, whose activity must be fine-tuned to ensure proper xylem development.

## 1 Introduction

Thermospermine, a structural isomer of spermine, is present in some bacteria and widely in the plant kingdom but not in fungi and animals (Takano et al., 2012). Exceptionally, its compound philanthotoxin 433 (PTX-433) is found in the venom of the digger wasp *Philanthus triangulum* (Eldefrawi et al., 1988). In vascular plants, thermospermine functions as a suppressor of xylem differentiation. In *Arabidopsis thaliana*, loss-of-function mutants of *ACL5*, which encodes thermospermine synthase, exhibit a dwarf phenotype with excessive xylem differentiation (Kakehi et al., 2008). When wild-type plants are grown at a high concentration of thermospermine, xylem differentiation and overall shoot growth is severely inhibited. Research into the specific mode of action of thermospermine has advanced through the isolation of suppressor mutants of *acl5*, named *sac*, which suppress the dwarf phenotype of *acl5* even in the absence of thermospermine (Imai et al., 2006). Analysis of the dominant suppressor mutant *sac51-d* revealed that thermospermine promotes the translation of the *SAC51* mRNA. *SAC51* encodes a bHLH protein and its mRNA contains multiple uORFs in its 5’ leader sequence. Typically, uORFs inhibit translation of the main coding sequence. In *sac51-d*, a nonsense mutation occurs within a uORF that is highly conserved across plant species and leads to increased translation efficiency of the bHLH protein even without thermospermine, thereby suppressing excessive xylem differentiation and restoring stem elongation. The causative genes for the dominant suppressors *sac52-d*, *sac53-d*, and *sac56-d* encode ribosomal proteins RPL10, RACK1, and RPL4, respectively, and all mutations were shown to enhance translation of the *SAC51* main ORF without thermospermine (Imai et al., 2008; Kakehi et al., 2015). Furthermore, the causative gene for *sac59*, *JMJ22*, is a homolog of *JMJD6*, which functions in RNA processing in animals. Its loss-of-function appears to stabilize the mRNAs of *SAC51* family members (Matsuo et al., 2022). Taken together with the known interaction of polyamines with RNA, these findings suggest that thermospermine acts on ribosomal RNAs to relieve the translational inhibition imposed by the conserved uORF.

*SAC51* forms a gene family along with *SACL1*, *SACL2*, and *SACL3*. Multiple mutant alleles of the conserved uORF of *SAC51*, *SACL1*, and *SACL3* have been isolated as suppressor mutants of *acl5* (Vera-Sirera et al., 2015; Nishii et al., 2023; Mutsuda et al., 2025). Conversely, the double knockout mutant of *SAC51* and *SACL3* protein coding regions shows insensitivity to thermospermine; xylem differentiation in the roots is not suppressed by 0.1 mM thermospermine treatment, although the quadruple knockout of all members exhibits mild morphological abnormalities compared with *acl5* (Cai et al., 2016). Expression of *ACL5* and *SACL3* is induced in xylem precursor cells by the bHLH heterodimers LONESOME HIGHWAY (LHW)-TARGET OF MONOPTEROS5 (TMO5) and LHW-TMO5 LIKE1 (T5L1) (Katayama et al., 2015; Vera-Sirera et al., 2015). These dimers are known as a critical regulator of auxin-dependent xylem formation (De Rybel et al., 2013). SACL3 has been shown to bind to LHW and prevent the formation of these dimers as a negative feedback factor of xylem development. It is thus likely that uORF mutants of *SAC51*, *SACL1*, and *SACL3* have a similar effect on this feedback regulation without thermospermine. Here we investigated the role of the remaining *SAC51* family member, *SACL2*, by generating uORF mutants. These mutants along with loss-of-function mutants of *LHW* were shown to be hypersensitive to thermospermine.

## 2 Materials and methods

### 2.1 Plant material and growth conditions

The Columbia (Col-0) accession of *Arabidopsis thaliana* was used as wild type. The mutants *acl5-1*, *sac51-d* (Imai et al., 2006), *sac51-1*, *sacl3-d* (*sac57-d*), *sacl3-1* (Cai et al., 2016), *sacl1-d* (*sac504-d*) (Mutsuda et al., 2025), have been described previously. *lhw* (SALK_079402C) and *lh13* (SALK_126132) were obtained from the Arabidopsis Biological Resource Center (www.arabidopsis.org). Multiple mutant combinations were generated by crosses and their genotypes were confirmed by PCR with gene-specific primers (**Supplementary Table S1**).

Plants were grown under 16-h light/8-h dark conditions at 22°C on rockwool bricks supplemented with vermiculite in the growth chamber. For seedling growth observation and RNA extraction, seeds were surface-sterilized with bleach solution containing 0.01% (w/v) Triton X-100 for 3 min, rinsed three times in sterile water, germinated and grown on 0.8% agar plates containing MS salts (Wako, Tokyo, Japan) and 1% sucrose at pH5.7. For examining the sensitivity to thermospermine, seeds were sown on MS agar plates containing 30 μM thermospermine (Santa Cruz, CA, USA). For examining the response of promoter-GUS fusions to hormones, each transgenic line was grown for 7 days on MS plates, transferred to MS solutions containing 1 μM 2,4-D, 1 μM kinetin, or 10 μM bikinin, and incubated for 24 h.

### 2.2 T-DNA construction and plant transformation

A transgenic line carrying the 990-bp *SAC51* promoter fused to the *GUS* gene was described previously (Ishitsuka et al., 2019). For constructing promoter-*GUS* fusions of *SACL1*, *SACL2*, and *SACL3*, each gene promoter was amplified by PCR with gene-specific primers (**Supplementary Table S1**), digested with restriction enzymes, and inserted upstream of the *GUS* reporter gene in pBI101 (Jefferson et al., 1987). For constructing CaMV 35S promoter-driven *SACL2* 5’-*GUS* fusions, the *SACL2* 5’ region was amplified by PCR (**Supplementary Table S1**) from wild-type and *sacl2-d1* genomic DNA and cloned into pBI121 (Jefferson et al., 1987). The resulting constructs were introduced into wild-type Col-0 plants by the floral dip method (Clough and Bent, 1998).

### 2.3 Genome editing

A CRISPR/Cas9 construct for the conserved uORF of *SACL2* was made by using pKIR vector as described (Tsutsui and Higashiyama, 2017) with a pair of oligonucleotides SACL2edit-F and SACL2edit-R (**Supplementary Table S1**). The construct was introduced into wild-type Col-0. Cas9-induced mutations were identified by PCR amplification of the *SACL2* target region using the primer pair, uL2-F and uL2-R (**Supplementary Table S1**), followed by sequencing. The edited lines in the *acl5* background were generated by crosses.

### 2.4 GUS assays

Fluorometric quantitative GUS assays and histochemical GUS staining were performed according to a standard protocol (Jefferson et al., 1987). Samples embedded in Technovit (Heraeus Kulzer, Wehrheim, Germany) were sectioned using a rotary microtome RM2245 (Leica Microsystems, Wetzlar, Germany).

### 2.5 Yeast two-hybrid assay

Y2H experiments were performed using the Matchmaker Gold Yeast Two-Hybrid System (Clontech, CA, USA). Full-length coding sequences of each gene were amplified by PCR with gene-specific primers (**Supplementary Table S1**) and cloned into pGADT7 and pGBKT7 vectors, respectively. Yeast transformants were tested for interactions in synthetic dextrose (SD) media lacking Leu, Trp, His, and adenine in the presence of 3 mM 3-amino-1,2,4-triazole (3AT).

### 2.6 qRT-PCR

Total RNA was extracted from whole seedlings by the SDS-phenol method followed by LiCl precipitation (Imai et al., 2006). Reverse-transcription was carried out using the PrimeScript RT reagent Kit (Takara, Kyoto, Japan) with the oligo(dT) primers. qPCR reactions were performed using KAPA SYBR FAST qPCR Kit (KAPA Biosystems, MA, USA) and the Thermal Cycler Dice TP760 (Takara) with gene-specific primers (**Supplementary Table S2**). *ACTIN8* (At1g49240) was used as an internal control.

## 3 Results and discussion

### 3.1 *SACL2* uORF mutants partially suppress the *acl5* phenotype

**Figure 1:**
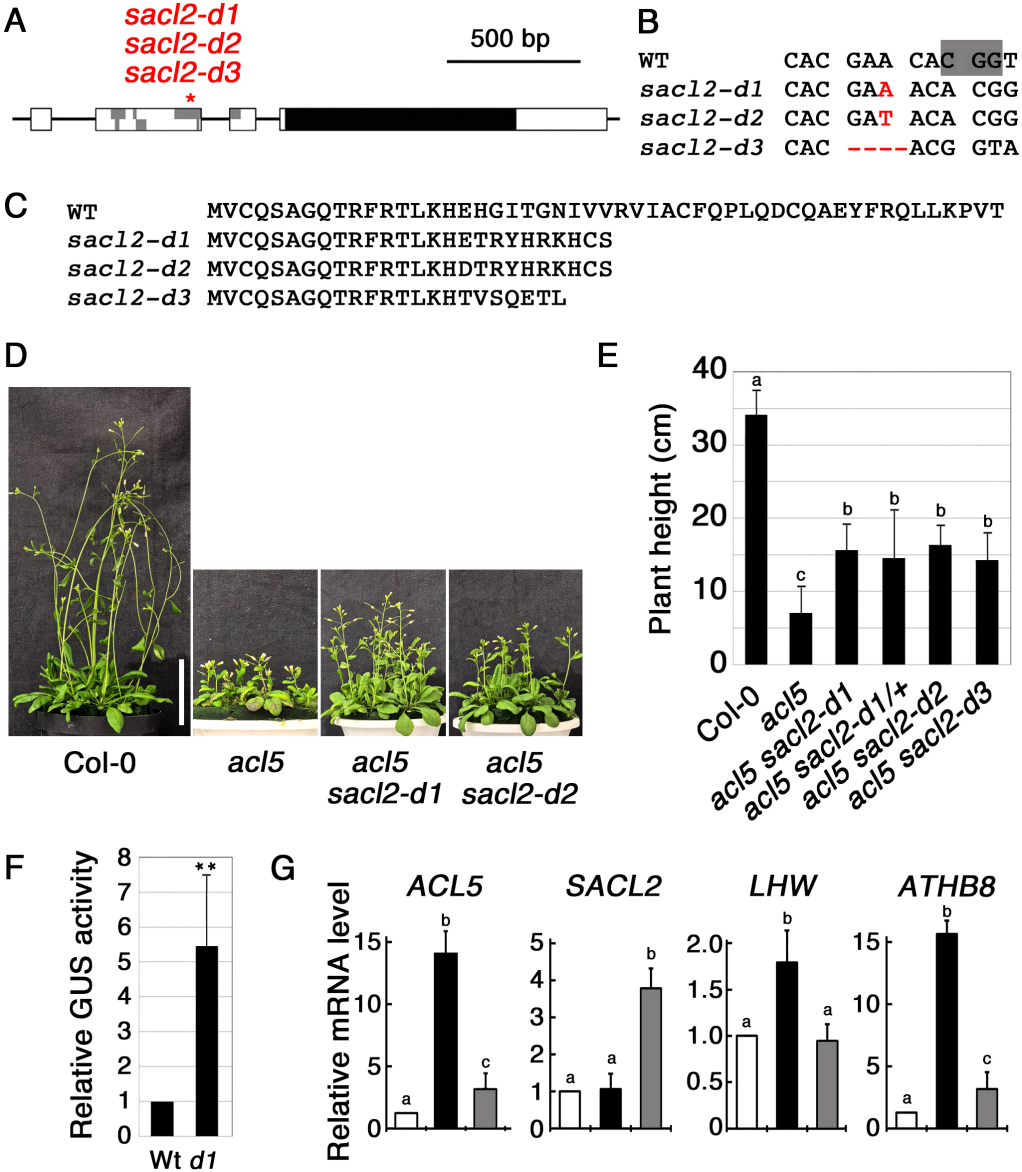
Characterization of uORF mutants of *SACL2*. **(A)** Genomic structure of *SACL2.* Boxes indicate exons, within which gray and black regions represent uORFs and a main ORF, respectively. An asterisk indicates the site edited by CRISPR/Cas9. **(B)** Alignment of the DNA sequence around the edited regions of *SACL2*. The PAM sequence is shaded in the wild-type (WT) sequence. Inserted and deleted bases are shown in red. **(C)** Alignment of the deduced amino acid sequence of the conserved uORF of *SACL2* in each allele. **(D)** Growth phenotype of 35-day-old wild-type Col-0, *acl5*, and double mutants (*acl5 sacl2-d1* and *acl5 sacl2-d2*). Bar = 5 cm. **(E)** Plant height comparison of 40-day-old plants. *d1/+* indicates a heterozygote of the *d2* allele. Error bars represent the SD (n = 10). Different letters indicate statistically significant differences at the 0.05 level by ANOVA/Tukey’s test. **(F)** Relative GUS activity derived from wild-type and *sacl2-d1* 5’-GUS fusions under the CaMV 35S promoter. Data are shown from a representative homozygous transgenic line carrying each construct. Error bar represents the SD (n = 5). Asterisks indicate the significant differences from wild-type (Student’s *t*-test, **p < 0.01). **(G)** Relative mRNA levels of *ACL5, SACL2, LHW* and *ATHB8* in 10-day-old seedlings of wild-type (white), *acl5* (black), and *acl5 sacl2-d1* (gray), examined by qRT-PCR. Different letters indicate statistically significant differences at the 0.05 level by ANOVA/Tukey’s test.

To examine whether disrupting the conserved uORF of *SACL2* suppresses the dwarf phenotype of *acl5*, we generated the mutants using the CRISPR-Cas9 method and isolated three alleles: two with 1-bp insertions and one with a 4-bp deletion (**Figures 1A, B**), all of which result in a premature stop codon within the uORF (**Figure 1C**). In the progeny of the cross with *acl5*, these mutant alleles were shown to partially suppress the dwarf phenotype of *acl5* in a dominant manner, and were named *d1*, *d2*, and *d3*, respectively (**Figures 1D, E**). In the following experiments, we used the *sacl2-d1* allele. To confirm that this mutation enhances its downstream translation, we placed the wild-type and *d1* mutant versions of the *SACL2* 5’ leader region between the CaMV 35S promoter and the GUS reporter gene. Several transgenic lines carrying these constructs were obtained. All lines carrying the *d1* construct showed significantly higher GUS activity than those carrying the wild-type construct. Results from a representative line homozygous for the GUS gene are shown in **Figure 1F**. The effect of *sacl2-d1* on gene expression was examined by quantitative RT-PCR. In 10-day-old seedlings, the transcript level of genes up-regulated in *acl5*, including *LHW* and *ATHB8*, which regulate *ACL5* (Baima et al., 2014; Katayama et al., 2015), as well as a point-mutated form of *acl5*, were reversed in *acl5 sacl2-d1* (**Figure 1G**). The increased accumulation of the mutant *acl5* transcript in *acl5* is due not only to the expansion of the expression domain in the xylem, but also to the release from the negative feedback regulation mediated by the *SAC51* family. In *sacl2-d1*, excess *SACL2* may suppress LHW function, thereby leading to a partial reduction in *ACL5* expression. This result is similar to other uORF mutants in the *SAC51* family and is consistent with their morphological phenotypes. In contrast, the level of the one-base-inserted *sacl2-d1* transcript was several times higher than the wild type, suggesting that the *sacl2-d1* transcript is stabilized by ribosomes translating the main coding sequence, even in the absence of thermospermine. At least *SAC51* and *SACL3* have been listed as a target of nonsense-mediated mRNA decay due to the presence of conserved uORFs (Rayson et al., 2012).

### 3.2 All members of the *SAC51* family are commonly expressed in the vasculature

**Figure 2:**
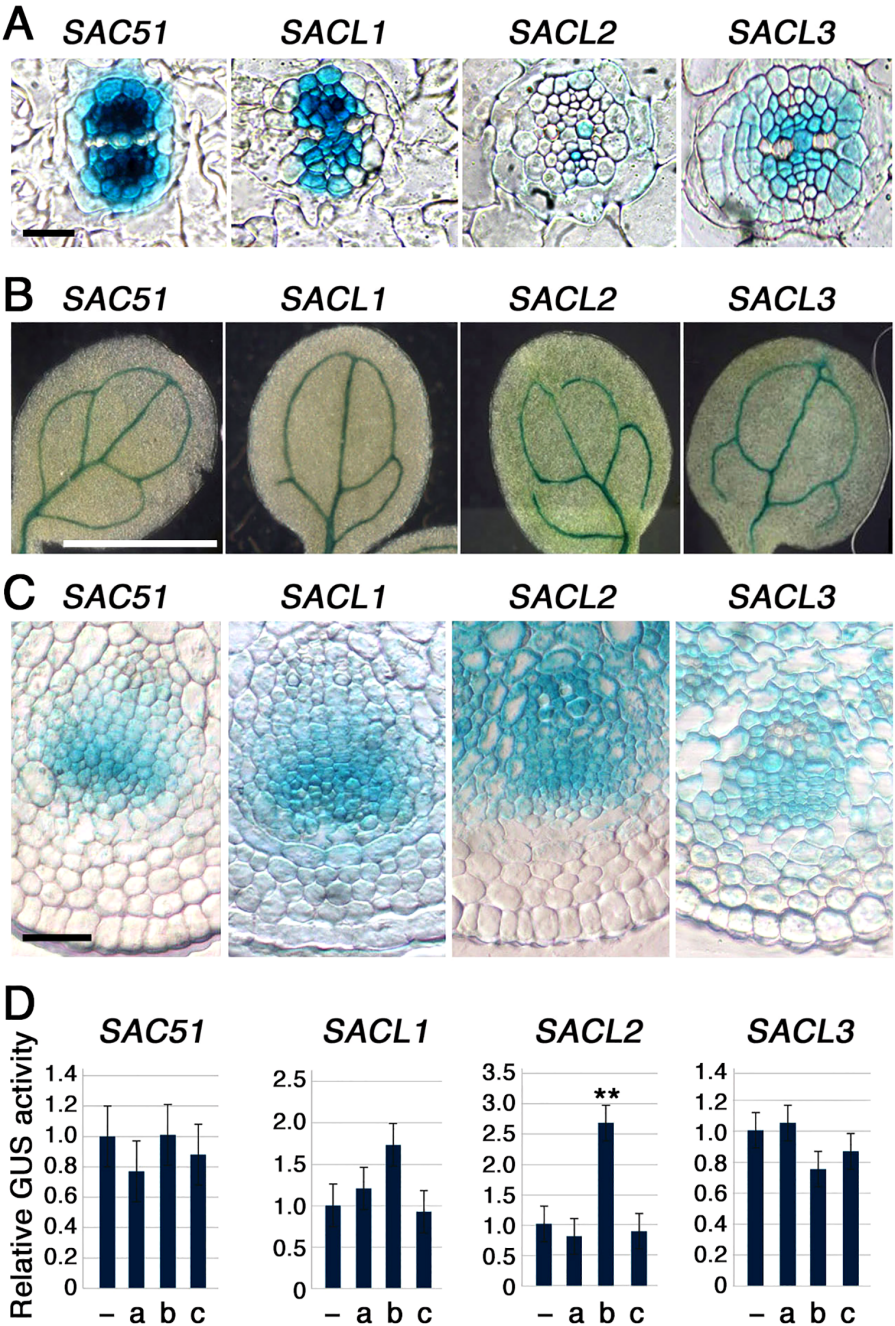
Promoter-GUS expression patters of the *SAC51* family. **(A)** Root sections of 5-day-old seedlings carrying each construct. Bar = 20 μm. **(B)** Cotyledons of 5-day-old seedlings carrying each construct. Bar = 1 mm. **(C)** Inflorescence stem sections of 35-day-old plant carrying each construct. Bar = 50 μm. **(D)** Relative GUS activity of 7-day-old seedlings carrying each construct treated with 1 μM 2,4-D (a), 10 μM bikinin (b), or 1 μM kinetin (c) for 24h. Error bar represents the SD (n = 5). Asterisks indicate the significant differences from wild-type (Student’s *t*-test, **p < 0.01).

Expression patterns of all *SAC51* family members were examined by using transgenic lines carrying each promoter-GUS fusion construct. In the root section, although the intensity varied, the entire central cylindrical vasculature, except for the protoxylem, was stained by all GUS constructs, with the highest staining observed in the procambium (**Figure 2A**). In cotyledons, staining was restricted to the veins in each construct (**Figure 2B**). In the inflorescence stem section, the GUS staining was consistently detected in the cambium and around the xylem vessels (**Figure 2C**). Together with the fact that uORF mutants of all *SAC51* family members can suppress the *acl5* phenotype, these results suggest that the regulatory systems governing the expression of all *SAC51* family genes are largely conserved. In addition to *SACL3*, *SACL2* has been shown to be regulated by LHW-TMO5 and LHW-T5L1 dimers (Katayama et al., 2015). The involvement of other members of the LHW and TMO5 families in the regulation of *SAC51* family expression still needs to be explored. We also examined the hormone response of these promoter-GUS fusions using 2,4-D, kinetin, and bikinin, an activator of brassinosteroid (BR) signaling, as these are particularly important for vascular differentiation. Although no significant increase was detected in the GUS activity after 24-h treatment of each transgenic seedling with 2,4-D and kinetin, the *SACL2* promoter was shown to be responsive to bikinin (**Figure 2D**), suggesting a possibility that BR-dependent induction was incorporated into the regulation of *SACL2* expression during molecular evolution.

**Figure 3:**
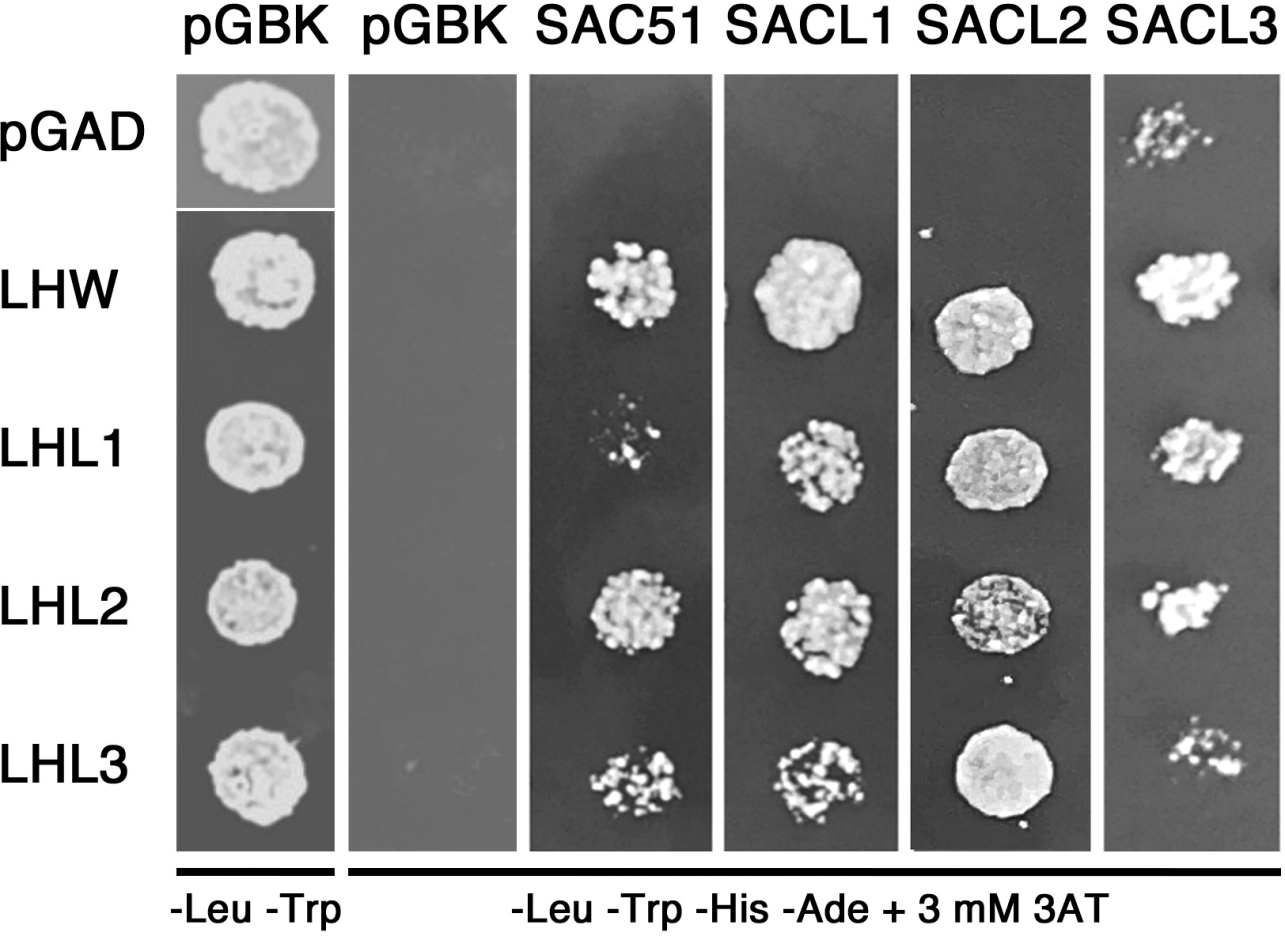
Y2H analysis of the interaction between the proteins of SAC51 and LHW families. Overnight cultures of yeast cells containing each plasmid construct were washed in water, plated on non-selective (-Leu -Trp) plates or selective (-Leu -Trp -His -Ade) plates supplemented with 3 mM 3AT, and incubated at 30°C for 3 days.

We further examined the physical interaction between SAC51 family proteins and LHW family proteins with Y2H. The result showed that all members of the SAC51 family can interact with all members of the LHW family (**Figure 3**). Interaction between SACL3 and LHW in Y2H has been shown previously (Ohashi-Ito and Bergmann, 2007; Vera-Sirera et al., 2015). In addition, interactions of LHW with SAC51, SACL1, and SACL2 were observed in bimolecular fluorescence complementation experiments (Katayama et al., 2015). These results support the possibility that suppression of *acl5* by *sacl2-d* mutants is also due to the functional repression of LHW and/or other members of the LHW family.

### 3.3 uORF mutants of the *SAC51* family are hypersensitive to thermospermine

**Figure 4:**
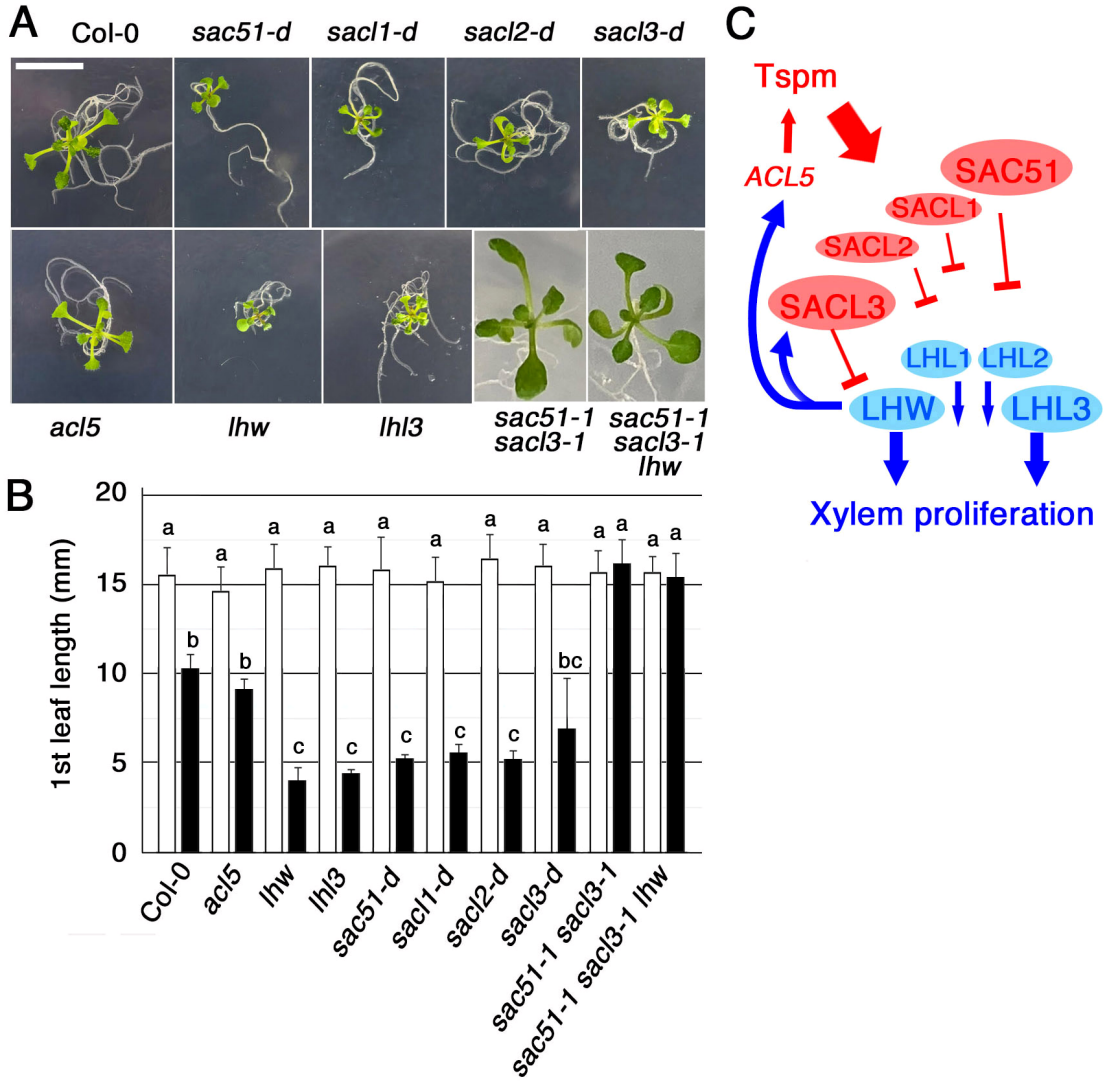
The seedling growth response to thermospermine. **(A)** Fourteen-day-old seedlings grown with 30 μM thermospermine. Bar = 1 cm. **(B)** The 1st leaf length of 14-day-old seedlings grown without (open bars) or with (filled bars) 30 μM thermospermine. Error bars represent the SD (n = 10). Different letters indicate statistically significant differences between genotypes and treatments at the 0.05 level by ANOVA/Tukey’s test. **(C)** A simplified model illustrating how the sensitivity to thermospermine (Tspm) is finetuned via interactions between the members of SAC51and LHW families.

Because the double knockout of *SAC51* and *SACL3* is highly insensitive to thermospermine (Cai et al., 2016), we examined whether uORF mutants of the *SAC51* family are hypersensitive to thermospermine. In the presence of 30 μM thermospermine, leaf expansion of all mutant seedlings, *sac51-d*, *sacl1-d* (*sac504-d*), *sacl2-d1* and *sacl3-d* (*sac57-d*), was severely inhibited compared to the wild type (**Figures 4A, B**). Given that SAC51 family proteins bind to LHW family proteins and interfere with their function in xylem cell proliferation, this hypersensitivity to thermospermine could be attributed to the functional inhibition of the LHW family. The tiny seedling phenotype is also reminiscent of higher-order loss-of-function mutants in LHW and TMO families (De Rybel et al., 2013; Ohashi-Ito et al., 2013; Vera-Sirera et al., 2015). We then examined the thermospermine sensitivity of loss-of-function mutants of *LHW* and *LHW LIKE3* (*LHL3*). *lhl3* shows no obvious phenotype but enhances the *lhw* phenotype in the root vascular development and causes a severe defect in the seedling growth in *lhw lhl3* (Ohashi-Ito et al., 2013). We confirmed that they are hypersensitive to thermospermine (**Figures 4A, B**). Furthermore, the insensitive phenotype of *sac51–1 sacl3–1* was unaffected in the triple mutant of *sac51–1 sacl3–1 lhw*. The result can be interpreted that, while the *SAC51* family acts as a brake on vascular xylem proliferation in contrast to the *LHW* family, which functions as an accelerator, xylem proliferation is not suppressed in *sac51–1 sacl3–1* even in the absence of *LHW*. This may be because *SACL1* and *SACL2* are not sufficient to repress the function of other *LHW* family members as accelerators, despite the loss of the primary one, *LHW*. In contrast, overproduction of one *SAC51* family member by its uORF mutation may be sufficient to enhance thermospermine sensitivity. A simplified scheme shown in **Figure 4C** indicates that the thermospermine sensitivity, and thus the degree of xylem proliferation, is regulated by interactions between all members of the SAC51 and LHW family proteins.

## 4 Conclusion

This study revealed that all members of the *SAC51* family are involved in mediating the response to thermospermine through interactions with all members of the LHW family. The partial suppression of the *acl5* phenotype by the uORF mutants of *SACL2* may be due to a lower level of main ORF translation and/or a weaker inhibitory effect on LHW function compared with the uORF mutants of other *SAC51* family members. While *SAC51* and *SACL3* are considered the main regulatory factors mediating the thermospermine response, the specific functions or biological significance of *SACL1* and *SACL2* remain unclear. They may play auxiliary roles under normal conditions but exhibit specific functions under limited growth conditions or developmental stages. The potential for functional specificity might depend on the particular pair of *SAC51* and *LHW* family. To further elucidate these relationships, it will be necessary to generate and analyze combinations of mutants from each gene family as well as from the TMO family.

## Data Availability

The original contributions presented in the study are included in the article/**Supplementary Material**, further inquiries can be directed to the corresponding author/s.
