# Supplementary material for: The response to thermospermine is fine-tuned by the balance between SAC51 and LHW family proteins in Arabidopsis thaliana
Source: Front Plant Sci. 2025 Sep 2;16:1654744. doi: 10.3389/fpls.2025.1654744 (PMC12436307; doi:10.3389/fpls.2025.1654744)
Supplement: Supplementary file 1 [file Table1.docx]

**Supplementary Table S1.** List of oligonucleotide primers for genotyping and vector construction

Primer name Primer sequence Purpose/ Remark

ACL5dCAPS-F ATGGGAGGAGGTGAAGGCTCTGCTGCTCGA Genotyping *acl5-1*/ dCAPS-XhoI

ACL5dCAPS-R TTTGTTACAGAAAGCATCGCTGTTAAC

L2d-F CATGCGGTGGTGGTTGTGTC Genotyping *sacl2-d*/ dCAPS-PvuI

L2d-RPvuI TAACTACAATGTTTCCTGTGATACCGCGAT

SACL2-5FXba TCTAGAAACTAATATCTCTCTGCTTC 35S-*SACL2* 5’-GUS fusion

SACL2-5RBam GGATCCAACACTAAACAAATCAACTATC

SACL1pro-FCla ATCGATGCTCCAACGTTTTTCACC Promoter-GUS fusion

SACL1pro-RXba TCTAGACGAATTCACGAAAGCTTG

SACL2pro-FHin gaagtcttataatgataatgaagcttg Promoter-GUS fusion

SACL2pro-RBam ggatccaacactaaacaaatcaacta

SACL3pro-FCla atcgattggcacaacttggaacc Promoter-GUS fusion

SACL3pro-RXba tctagaaaacccgtgcaaacgta

SACL2edit-F ATTG CGTACACTCAAACACGAACA *SACL2*-editing vector construction

SACL2edit-R AAAC TGTTCGTGTTTGAGTGTACG

uL2-F GATACTCTGTTTTGGGAGAAG Screening for edited mutants

uL2-R CCCATCCAACACTAAACAAATC

SAC51-1F TGTTCGTTGGTCACTTTCATCT Genotyping *sac51-1*

SAC51-1R TCTTAGCAGTTTAGCTCAAGG R + pBI-LB: *sac51-1* T-DNA

SACL3-1F TCTATTGAATGTTCTGTCTTGTTT Genotyping *sacl3-1*

SACL3-1R GAAGAGGATACTTCTTGCTC R + pBI-LB: *sacl3-1* T-DNA

LHW-F CCAAGAAGCAGAGAAAAGAAA Genotyping *lhw*

LHW-RR AGTCTTCTAGGATTCGAGCT F + pBI-LB: *lhw* T-DNA

pBI-LB AACCAGCGTGGACCGCTTGCTG Detection of T-DNA

SAC51-FNde CATATGCCTCTGGATAAGAGGCAAC Y2H vector construction

SAC51-RBam GGATCCTTATCTGTCTGCAGATTTC

SACL1-FNde CATATGCCTCTTGATACCAAACAG Y2H vector construction

SACL1-RBam GGATCCAATGGTTATTCAAACCCTTTG

SACL2-FNde CATATGGGACAAGATCGTGGGTTTG Y2H vector construction

SACL2-RBam GGATCCATGCTTCTCGATTTTTCGCTTC

SACL3-FEco GAATTCATGCAGAACAATCAGTTTCCTC Y2H vector construction

SACL3-RBam GGATCCAAGATTGGTTTGAGAAATGTC

LHW-FNco CCATGGGAGTTTTACTAAGAGAAGC Y2H vector construction

LHW-RBam GGATCCATTGAACAGCCACCAGTAAC

LHL1-FNde CATATGGGTTATACCTTGCAACAGATAC Y2H vector construction

LHL1-RBgl AGATCTGTTTGTAGCCTTGGGTTG

LHL2-FNde CATATGGGTTCTACTTCTCAAGAG Y2H vector construction

LHL2-RBgl AGATCTTACCCCTTCTCATTGGCCT

LHL3-FNde CATATGGGTTCAGAGTATAAGCATATA Y2H vector construction

LHL3-RPst CTGCAGATAATAAATCATCATGTTTGGTG

**Supplementary Table S2.** List of oligonucleotide primers for qRT-PCR

Primer name Primer sequence Gene ID

ACT8-F GTCGTACAACCGGTATTGTG At1g49240

ACT8-R TCTCTTGCTCGTAGTCGACAG

ACL5-F ACCGTTAACCAGCGATGCTTT At5g19530

ACL5-R CCGTTAACTCTCTCTTTGATTC

SACL2-F GTAGTTGTGACAACACAAG At5g50010

SACL2-R ACAACCTGATCCCTTTGTAG

LHW-F TCCAAGTCGAGATGTTATGC At2g27230

LHW-R CGGTTTATTAAACCGGAGCA

ATHB8-F AGCGTTTCAGCTAGCTTTTGAG At4g32880

ATHB8-R CAGTTGAGGAACATGAAGCAGA
